# Generation and Characterization of Vascular Smooth Muscle Cell Lines Derived from a Patient with a Bicuspid Aortic Valve

**DOI:** 10.3390/cells5020019

**Published:** 2016-04-21

**Authors:** Pamela Lazar-Karsten, Gazanfer Belge, Detlev Schult-Badusche, Tim Focken, Arlo Radtke, Junfeng Yan, Pramod Renhabat, Salah A. Mohamed

**Affiliations:** 1 Department of Cardiac and Thoracic Vascular Surgery, University of Luebeck, D-23538 Luebeck, Germany; pamela.lazar-karsten@uksh.de (P.L.-K.); detlev.schult-badusche@uksh.de (D.S.-B.); junfeng.yan@uksh.de (J.Y.); pramod.renhabat@medizin-uni-luebeck.de (P.R.); 2 Center of Human Genetics and Genetic Counselling, University of Bremen, D-28359 Bremen, Germany; belge@uni-bremen.de (G.B.); focken@uni-bremen.de (T.F.); arlo.radtke@gmx.de (A.R.)

**Keywords:** bicuspid aortic valve, vascular smooth muscle cell, WG-59 cell line, losartan

## Abstract

Thoracic aortic dilation is the most common malformation of the proximal aorta and is responsible for 1%–2% of all deaths in industrialized countries. In approximately 50% of patients with a bicuspid aortic valve (BAV), dilation of any or all segments of the aorta occurs. BAV patients with aortic dilation show an increased incidence of cultured vascular smooth muscle cell (VSMC) loss. In this study, VSMC, isolated from the ascending aorta of BAV, was treated with Simian virus 40 to generate a BAV-originated VSMC cell line. To exclude any genomic DNA or cross-contamination, highly polymorphic short tandem repeats of the cells were profiled. The cells were then characterized using flow cytometry and karyotyping. The WG-59 cell line created is the first reported VSMC cell line isolated from a BAV patient. Using an RT^2^ Profiler PCR Array, genes within the TGFβ/BMP family that are dependent on losartan treatment were identified. Endoglin was found to be among the regulated genes and was downregulated in WG-59 cells following treatment with different losartan concentrations, when compared to untreated WG-59 cells.

## 1. Introduction

Vascular smooth muscle cells (VSMCs) are of mesodermal origin and are present in various organs (essential parts of the cardiovascular system, the respiratory system, the gastrointestinal tract and the urinary tract). The VSMC phenotype is diverse [1]. In normal mature blood vessels, the predominant phenotype is the quiescent or differentiated (contractile) phenotype, which regulates blood vessel diameter and blood flow [2]. This phenotype can switch into the proliferative, migratory and synthetic type, which is crucial for repair in response to injury and the synthesis of extracellular matrix proteins, such as fibroblast growth factor, epidermal growth factor, platelet-derived growth factor, transforming growth factor beta (TGFβ), vascular endothelial growth factor and angiotensin II (AngII) [3]. VSMCs are the main component of the aortic media, and their dysfunction plays an important role in many different arterial diseases [4].

The loss of VSMCs is a characteristic of degenerative remodeling within the medial layer of thoracic aortic aneurysms and dissection (TAAD) tissue. Moreover, patients with bicuspid aortic valve (BAV) and aortic aneurysms exhibit VSMC alpha actin gene mutations (*ACTA2*, mapped to chromosome 10q) [5,6]. In addition, a failure in switching from a synthetic to contractile VSMC phenotype generates intimal vascular lesions. VSMCs of patients with BAV are less mature than those of tricuspid aortic valve (TAV) patients [7]. There is a significant difference between the aortic wall structure in BAV patients compared to that in TAV patients, who present a thinner wall, lower expression of typical maturation markers, such as SM22 α, calponin, α smooth muscle actin and nearly no expression of smoothelin. BAV represents a high risk factor of TAAD. Patients with BAV are more frequently affected by TAAD and at a younger age [8,9].

VSMCs of the aortic valve and ascending aorta share a common embryonic origin, the cardiac neural crest [10,11]. Thus, analyzing the diseased ascending aorta can provide useful information about malformations of the aortic valve. The dilation of the ascending aorta represents 43% of arterial diseases with an upward trend. Due to the high mortality and lack of symptoms, it is a special challenge to physicians, internists and cardiac surgeons. Since patients with BAV show increased susceptibility to the development of ascending aortic dilation, studying the aortic wall structure may render interesting insights. VSMC culture may provide useful help for this matter.

We used VSMCs isolated from a patient with BAV/TAAD and performed a Simian virus 40 (SV-40) transfection to generate an immortalized VSMC cell line. We then characterized these cells and performed spectral karyotyping (SKY) to eliminate potential contamination by foreign DNA.

Since the TGFβ pathway plays an important role in the pathogenesis of TAAD, we analyzed the effect of losartan on the expression of 84 genes related to the TGFβ pathway in VSMCs. The disease Marfan syndrome (MFS), a connective tissue disorder, is caused by a mutation in the *Fibrillin-1* gene. This mutation leads to overexpression of TGFβ signaling in MFS. Loeys–Dietz syndrome (LDS), another disease with particularly strong predisposition for arterial aneurysm, shows decreased TGFβ signaling. This leads to the assumption that TGFβ dysregulation may promote the aneurysmal process in the aorta, thus arousing interest in further investigations of TGFβ. In the present study, the AngII type 1 receptor (AT_1_R) blocker losartan was used as a potential TGFβ signaling inhibitor. Losartan reduces aortic growth and blunts TGFβ signaling in the aortic media of fibrillin-1-deficient mice, thereby indicating an impact of the renin-angiotensin system in thoracic aortic aneurysm [12].

## 2. Material and Methods

### 2.1. Cell Culture of Primary VSMCs

A 46-year-old male patient was diagnosed with BAV and fusion of the right and non-coronary cusps. He was admitted to the hospital to undergo aortic valve replacement due to Grade 3 pre-valve stenosis. Ascending aorta tissue was removed during surgery; VSMCs were isolated out of the tunica media for cell culture. The vascular smooth muscle sample was minced and treated with 0.26% collagenase (250 U/mL, Serva; Heidelberg, Germany) at 37 °C for 3–4 h. Following centrifugation, the pellet was resuspended in culture medium (TC199 supplemented with Earle's balanced salt solution, 20% fetal bovine serum (FBS), 200 IU/mL penicillin and 200 µg/mL streptomycin and incubated at 37 °C, 5% CO_2_). The monolayer culture was passaged by standard trypsin dispersion and resuspended in culture medium.

### 2.2. Generating the WG-59 Cell Line from Primary VSMC Culture

To generate a human ascending aorta vascular smooth muscle cell line with an extended life span, primary smooth muscle cells isolated from the biopsy material were transfected with a mammalian expression vector containing genes encoding the SV-40 early region, according to Kazmierczak *et al.* [13]. Initial foci of transformed cells appeared 26 days post-transfection.

### 2.3. Fluorescence in Situ Hybridization Mapping of the SV-40 Early Region in VSMCs after Transfection

FISH studies were performed on pre-banded metaphase chromosomes from transformed muscle cells. For hybridization, SV-40 plasmid DNA was used with DNA probes approximately 7.5 kb in length, as described previously [13]. The hybridization procedure was performed according to the manufacturer’s instructions (Roche Diagnostics; Mannheim, Germany). The DNA probes were treated with digoxigenin (DIG, Roche Diagnostics; Mannheim, Germany) and dissolved in hybridization media followed by overnight incubation in a moist chamber at 37 °C. Labeled probes were detected using anti-digoxigenin–fluorescein isothiocyanate conjugates (FITC, Roche Diagnostics; Mannheim, Germany). Chromosomes were counterstained with propidium iodide. In total, 20 metaphase events were scored for analysis.

### 2.4. Immunofluorescence for Large T-Antigen in the WG-59 Cell Line 

Large SV-40 T-antigen expression was analyzed according to Kazmierczak *et al.* [13]. Approximately 5 × 10^4^ VSMCs were plated onto cover slips and incubated in TC199 culture medium supplemented with 20% (v/v) FBS for 48 h. Cells were washed with phosphate-buffered saline (PBS) and fixed with methanol and acetone (10 min each at −20 °C). Cells were incubated for 30 min with mouse anti-SV40 large T-antigen, washed in PBS and incubated with FITC-labeled goat anti-mouse IgG (Merck Biosciences; Schwalbach, Germany) for another 30 min.

### 2.5. Chromosome Analysis with Spectral Karyotyping of the WG-59 Cell Line

For chromosome analysis, we used exponentially-growing cultures of transformed VSMCs. After a 24-h growth period, metaphase chromosome spreads were prepared using colcemid (0.06 µg/mL for 40 min) in order to arrest the cultured cells during mitosis. A hypotonic solution (1:6 ratio of culture medium in aqua bidestllata. and a fixative (3:1 ratio of methanol and acetic acid) were sequentially applied. Finally, the chromosome suspension was dropped onto glass slides. The chromosomes were GTG-banded according to routine techniques. The karyotype description followed the International System for Human Cytogenetic Nomenclature 2013 (ISCN). SKY was performed according to the manufacturer’s protocol (Applied Spectral Imaging (ASI), Migdal Ha’Emek, Israel). To describe the karyotype, 12 metaphase spreads were acquired with an Axioskop 2 plus microscope (Carl Zeiss, Göttingen, Germany) and analyzed using the SkyView Software. A composite karyotyping was done according to ISCN 2013.

### 2.6. Highly Polymorphic Short Tandem Repeat Profiling

DNA was isolated from patient tissue and WG-59 using the QIAamp DNA mini kit (Hilden, Germany) according to the manufacturer’s protocol. DNA concentration was measured using the NanoDrop instrument (Wilmington, NC, USA). To screen the cells against cross-contamination and in order to authenticate them as a human cell line originating from the donor’s material as the donor tissue, short tandem repeat (STR) typing was performed. DNA profiling was carried out using 8 different and highly polymorphic STR loci. Furthermore, we tested the samples for the presence of mitochondrial DNA sequences from rodent cells, such as mouse, rat, Chinese and Syrian hamster, according to the manufacturer’s protocol (DSMZ, Braunschweig, Germany).

### 2.7. Characterization of the WG-59 Cell Line

The WG-59 cell characterization was performed by flow cytometry at the 20th passage. In brief, 100 µL of 1 × 10^6^ cells were incubated for 20 min at 4 °C in BD Cytofix/Cytoperm (BD Bioscience, Heidelberg, Germany) in order to fix and the cells and permeabilize the cell membrane. Cells were then washed with 10% BD Wash/Perm Solution (BD Bioscience) and incubated with antibodies against α-actin (R&D Systems, Minneapolis, MN, USA), myosin heavy chain (R&D Systems, Minneapolis, MN, USA), calponin (Acris, San Diego, CA, USA) and smoothelin (Acris, San Diego, CA, USA) in a total volume of 50 µL for 30 min. Cells were washed again in 10% BD Wash/Perm Solution and were resuspended in 300–400 µL BD Staining Buffer (BD Bioscience). The measurement was performed on a benchtop analyzer LSR II (Becton Dickenson, Heidelberg, Germany). FlowJo software (Tree Star, Inc., Ashland, OR, USA) was used for data analysis. To exclude non-specific binding, mouse IgG1 and IgG2a isotype controls (BioLegend, London, UK) were used.

### 2.8. Collagen-Based Cell Contraction Assay

The contractility of the WG-59 cells at a passage of 50 was tested by subjecting the cells to a collagen-based contraction analysis, using the cell contraction assay (Cell Biolabs Inc. San Diego, CA, USA) according to the manufacturer’s protocol. Here, the cells were suspended in the DMEM media provided, with a density of 5 × 10^6^ cells per mL. The collagen lattice was prepared by mixing 1 part of the cell suspension with 4 parts of cold collagen gel solution (prepared with the supplied kit solutions). To polymerize the collagen matrix, 0.5 mL of the cell-collagen mixture were then added to each well in a 24-well plate and incubated for 1 h at 37 °C. After polymerization, 1 mL of the culture medium was added to each collagen gel lattice. The cell culture was incubated for 2 days. To initiate contraction, the collagen gel was gently released from the side of the culture dishes with a sterile spatula. The size of the matrix was measured at the beginning and end of the assay.

### 2.9. TGFβ Pathway and Data Analysis

The WG-59 cell line was grown to 80% confluency and incubated in serum-free media for 24 h. Losartan (1 µM) was added to the cells for 24 h. Cellular RNA was extracted with TRIzol (Life Technologies, Darmstadt, Germany). RNA was reverse transcribed into cDNA using theSuperScript II-Kit (Life Technologies, Darmstadt; Germany), and expression of 84 genes related to the TGFβ pathway was quantified using the RT^2^ Profiler PCR Array according to the manufacturer’s protocol (Qiagen, Hilden, Germany). Three independently-treated samples and three controls were measured using the RT^2^ Profiler PCR Array.

Data analysis was performed with RT^2^ Profiler PCR Array Data Analysis Version 3.5. The sample results were normalized to GAPDH and α-actin. Samples treated with losartan were compared to cells without treatment (control group). Genes with at least a two-fold change in expression and a *p*-value < 0.05 were considered significantly regulated.

### 2.10. Single Gene Expression Analysis

To confirm the TGFβ pathway analysis results and to further investigate the effect of losartan in various concentrations, the expression of *endoglin* and *CDC25A* was measured by single real-time RT-PCR. SYBR Green was used to detect expression changes dependent on different losartan concentrations (1, 10 and 100 µM) and compared to a non-treated control cell culture. As an internal control for normalization, 18S RNA was used. Gene expression analysis was performed using the Applied Biosystems 7000 Real-Time PCR System (Applied Biosystems by Life Technologies, Darmstadt, Germany).

## 3. Results

### 3.1. Generating and Characterizing the WG-59 Cell Line

**Figure 1:**
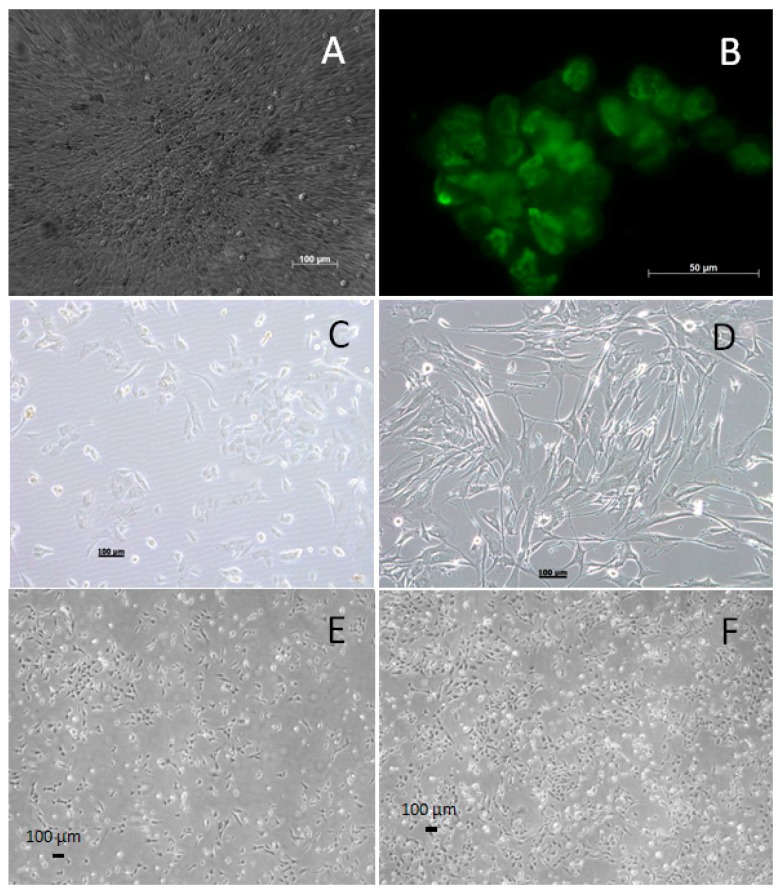
(**A**) Initial foci of transformed cells 26 days post-transfection in the bright field microscope. Foci are clearly distinguishable among the cultured and transfected cells. (**B**) GFP-labeled T-antigen expression in transfected cells. (**C**) Immortalized confluent WG-59 in comparison with non-transfected, primary confluent VSMCs; 10-fold enlargement (**D**). (**E**) WG-59 cell culture, third day of 20. Passage; 10-fold enlargement. (**F**) WG-59 cell culture, sixth day of 20. Passage; 10-fold enlargement.

The WG-59 cell line was generated from VSMCs of the tunica media of an ascending aorta. Immortalization was performed after SV-40 transfection. Initial foci of transformed cells appeared 26 days post-transfection (Figure 1A). Cells showed altered morphology, smaller cell size, loss of contact inhibition, foci formation and T-antigen expression (Figure 1B). Transformed cells showed higher proliferation capacity when compared to non-transformed cells. While non-transformed cells had a maximum lifetime of 15 passages, we were able to cultivate the transformed cells up to the 70th passage. Changes of the morphology and proliferation were monitored daily under the microscope (Figure 1C).

### 3.2. Cytogenic Characteristics and DNA Profile of the WG-59 Cell Line

**Figure 2:**
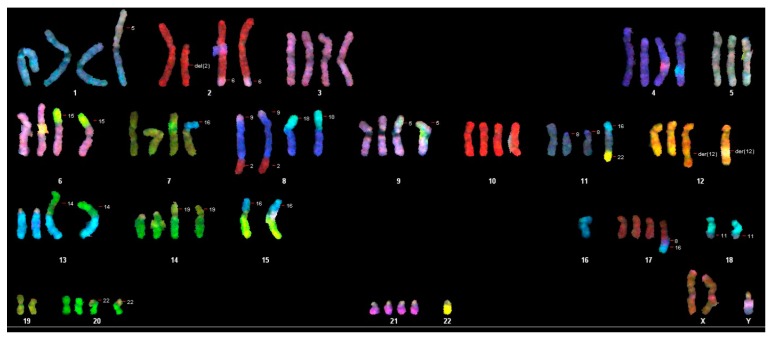
Illustration of detected chromosomes in WG-59 immortalized cultured cells in display colors, showing a pseudotriploid cell line with several aberrations.

To cytogenetically characterize the cell line, we investigated 60 metaphases (55th passage) where we found the largest (92) and the least (70) number of chromosomes (Table 1). The average number of chromosomes per metaphase is 76.6, so each chromosome should be present in three to four copies. We karyotyped 17 of the 60 metaphases and found the loss of both Y-chromosomes in ten metaphases. In the other five karyotyped metaphases, only one Y-chromosome was present. In addition to different translocations and deletions of several chromosomes, the loss of autosomal chromosomes 2, 3, 4, 8, 9, 10, 13, 15, 17, 18, 19, 20 was observed in most metaphases. The spectral karyotyping revealed a pseudotriploid karyotype with a chromosome count of 70–91. Several aberrations were present in duplicates and clonal numbers. Single cell aberrations could be detected in nearly every metaphase (Figure 2).

**Figure 3:**
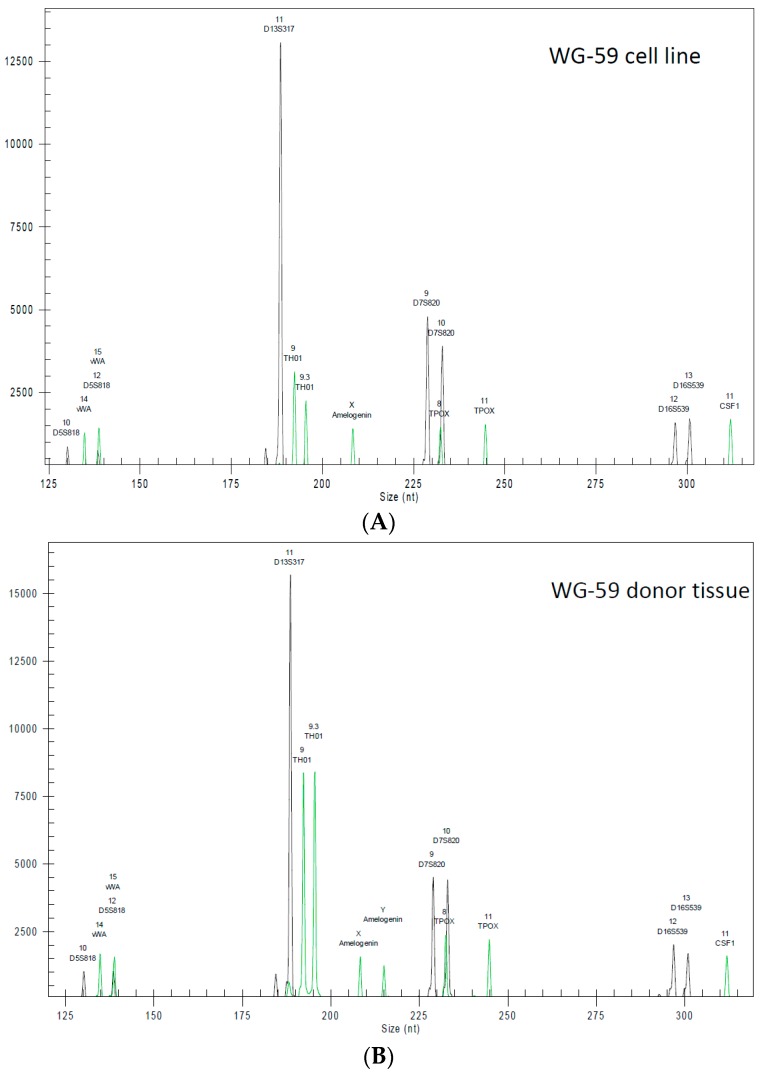
Electropherogram of the short tandem repeat (STR) profiling of (**A**) the WG-59 cell line and (**B**) the WG-59 donor tissue.

The sample WG-59 cell line was derived from WG-59 donor tissue, with full authentication stated (Table 2). Furthermore, using a detection limit of 1:10^5^, no mitochondrial sequences from mouse, rat or Chinese and Syrian hamster cells could be detected in WG-59 samples. The samples were derived from pure human cell cultures. Generated STR profiles of the WG-59 cell line showed no match with any reference cell line (Figure 3), as indicated by the search of the databases of cell banks ATCC (USA), Japanese Collection of Research Bioresources Cell Bank (Japan), RIKEN (Japan), Korean Cell Line Bank (Korea), Wistar Institute (USA), European Collection of Authenticated Cell Cultures (UK) and Deutsche Sammlung von Mikroorganismen und Zellkulturen (DSMZ, Germany). The WG-59 cell line shows a missing Y-chromosome in the STR analysis (Table 2).

### 3.3. Flow Cytometry Analysis of the WG-59 Cell Line

**Figure 4:**
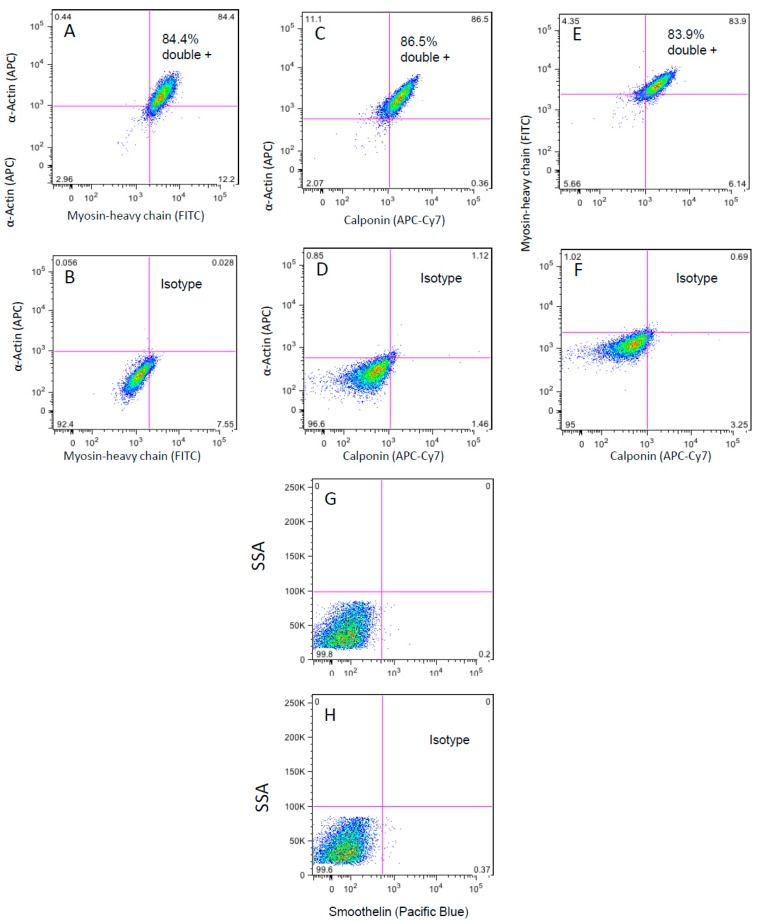
Purity analysis of isolated VSMCs with flow cytometry showing α-actin and myosin heavy chain-positive cells at the 20th passage and 80%–90% confluency. (**A**) Isotype control; (**B**) WG-59 cells; (**C**–**D**) purity analysis of isolated VSMCs with flow cytometry showing α-actin- and calponin-positive cells at the 20th passage and 80%–90% confluency; (**E**–**F**) purity analysis of isolated VSMCs with flow cytometry showing myosin heavy chain- and calponin-positive cells at the 20th passage and 80%–90% confluency; (**G**–**H**) purity analysis of isolated VSMCs with flow cytometry showing smoothelin and the isotype control of WG-59 cells at the 20th passage and 80%–90% confluency. Histogram plot of Smoothelin expression (G). Isotype control in pseudo color plot (H).

The purity of isolated cells was analyzed by flow cytometry. The cells expressed the typical VSMC proteins, e.g., α smooth muscle actin and myosin heavy chain. As shown in Figure 4A–C, 84.4% of SMCs were double positive for α smooth muscle actin and myosin heavy chain; 86.5% were positive for α smooth muscle actin and calponin; and 83.9% for myosin heavy chain (SM22) and calponin; whereas negative cells accounted for 2.98%, 2.07% and 5.66%, respectively. WG-59 cells showed an almost lack of smoothelin expression, as already observed by Grewal *et al.* [7] and declared typical for VSMC isolated from BAV patients (Figure 4G,H).

### 3.4. Cell Contraction Assay

Using the cell contraction assay, we aimed to visualize the contraction ability of WG-59 cells. The size of the collagen gel matrix was measured at the beginning and end of the assay. However, no size change could be detected at any time during the assay incubation period of seven days after stress release.

### 3.5. Gene Expression Analysis of the TGFβ Pathway

**Figure 5:**
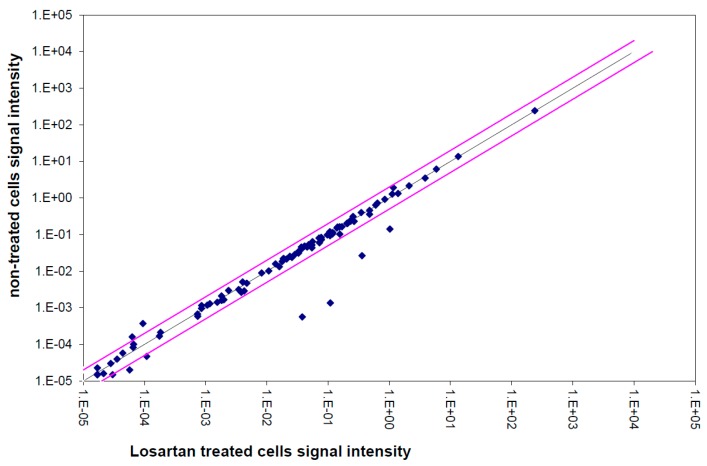
Correlation between the signal intensities of TGFβ-pathway genes. Expression analysis in losartan-treated and untreated cells. Cells treated with losartan are plotted against untreated cells. The scatter plot shows a positive tendency with a strong correlation between the two samples.

Various gene expressions of the TGFβ pathway were significantly different in losartan-treated VSMCs compared to untreated cells. However, the expression differences were low; none of the significant changes were over two-fold (Figure 5). The scatter plot showed a positive and strong correlation between losartan-treated cells and non-treated culture. Among the genes showing the most significant expression changes were *endoglin* (1.14-fold downregulation after losartan treatment; *p* = 0.0052) and *CDC25A* (1.24-fold downregulation after losartan treatment; *p* = 0.0008, Table 3).

**Figure 6:**
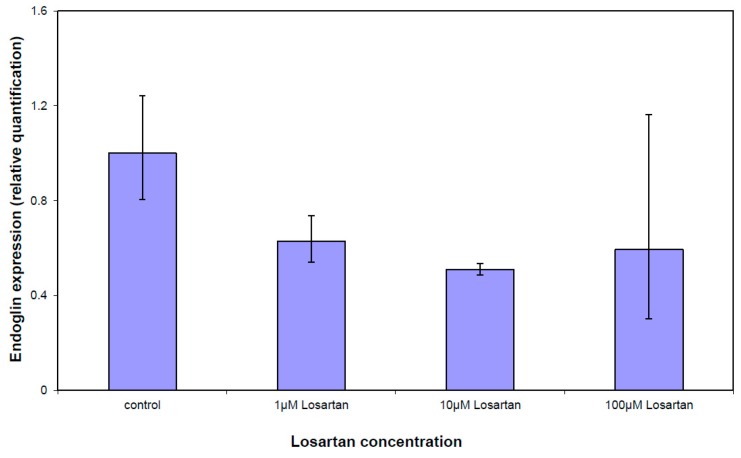
Single real-time RT-PCR of endoglin after WG-59 treatment with increasing losartan concentrations. Increasing the losartan concentration could not downregulate endoglin expression significantly.

*Endoglin* and *CDC25A* expressions were measured after treating cells with three different concentrations of losartan (Figure 6). Single real-time RT-PCR analysis confirmed gene expression array results. Endoglin was downregulated in both measurements. Increasing the losartan concentration caused downregulation of *endoglin* expression, but not significantly.

## 4. Discussion

TAAD is estimated to occur in three cases per 100,000 individuals per year and is a major cause of death [14]. Some publications define TAAD by medial degeneration characterized by VSMC loss, fragmentation and depletion of elastic fibers, as well as accumulation of proteoglycans and glycosaminoglycans within cell-depleted aorta tunica media areas [15]. Due to the role of VSMCs in TAAD, it is crucial to investigate the potential contribution of VSMC to TAAD development. An immortalized VSMC cell line could be an essential and powerful tool for this investigation.

In this study, we generated an immortalized VSMC cell line, WG-59, isolated from the aorta of a BAV patient. The WG-59 cell line showed a missing Y-chromosome in STR analysis. The phenomenon of uniparental disomy is sometimes observed during cell line establishments and is most probably due to loss of function mutations within DNA repair genes. Connected to bottlenecking outgrowths of sub-lines, these modifications can become visible.

Infection of human cells by SV-40 alters the cells and leads to characteristic changes. Transformation can be defined by expression of SV-40 large T-antigen, loss of contact inhibition, enhanced growth potential and growth in low-serum media [16]. Immortalized cell cultures usually enter a “crisis” characterized by reduced proliferation, loss of attachment to the flask and the development of multinucleated or giant cells [17]. Cultures surviving this crisis show very high growth capacity and provide an excellent working base for long-term experiments. To confirm our immortalized WG-59 cell culture containing VSMC, cell shape was observed daily by microscopy, and flow cytometric measurements were performed.

Cytogenetic studies of WG-59 cells showed polyploid karyotypes with complex rearrangements, loss of different autosomal chromosomes and both Y-chromosomes. The karyotype instability of the Y-chromosomes in cells is typical for the loss of the small chromosomes in rapidly-proliferating cells similar to immortalized SV-40 cell lines [13]. This might be the reason for the alteration in the cytogenic profile of the cell culture, e.g., loss of the amelogenin marker in STR analysis. The telomeric associations of the acrocentric chromosomes characteristic for SV-40 transformed cells were also found [18].

The expression of typical VSMC α smooth muscle cell actin, myosin heavy chain and calponin was detected in more than 95% of the cells (single- and double-positive cells), indicating that the culture consisted mainly of VSMCs. Other than VSMCs of TAV patients, VSMCs isolated from BAV patient tissue lack smoothelin expression, as shown by Grewal *et al.* [7] and confirmed in the present study.

The VSMCs phenotype varies from quiescent and contractile to proliferative and synthetic to execute a diversity of functions. Unlike the contractile phenotype, the synthetic phenotype possesses a high proliferative index and a low expression of contractile apparatus proteins [19]. *In vitro*, the synthetic VSMC phenotype tends to grow in a hill and valley morphology, which has been observed in previous studies of WG-59 VSMC cultures conducted by this group [20]. Thus, the hill and valley morphology observed in these cultured cells may be due to the fact that WG-59 cells are of a synthetic and proliferative phenotype and therefore express only a low amount of contractile proteins. This would explain the fact that no observable change in the collagen matrix size or in the contractility of the WG-59 cell culture in the cell contraction assay was detected. This would explain the fact that we were not able to observe any collagen size change and therefore contractility of WG-59 in the cell contraction assay.

The exact mechanisms leading to aortic aneurysms in the ascending aorta of bicuspid aortic valve (BAV) are poorly understood. It is unclear whether aortic changes in patients with BAV (large aortic diameter, reduced arterial elasticity and increased degeneration of the aortic media layer) relate to hemodynamic forces due to aortic valve dysfunction or whether these abnormalities result from an aortic wall weakness caused by genetic defects [21]. Several BAV studies have revealed high rates of VSMC apoptosis [22,23], similar to those observed in MFS, even in the non-dilated aorta [24]. MFS showed overexpression of TGFβresulting from a mutation of the *FIBRILLIN-1* gene [25]. On the other hand, in tissues obtained from patients with LDS, there is an apparent paradoxical increase in canonical TGFβ signaling with an increased expression of SMAD2, CTGF and collagen. The opposing mechanisms underlying this unexpected and paradoxical effect are unclear. However, the situation is the same in LDS as that in BAV-associated aortopathy. Losartan, an AT_1_R antagonist, was suggested to reduce aortic dilation rates at least in a small pediatric cohort of MFS patients [26]. In the present study, the effect of losartan on the TGFβ signaling pathway was investigated due to the similarity of aortic dilation to those in MFS and LDS.

In addition to the genes *TGFβ1*, *TGFβR1* and *SMAD3,* the gene encoding *auxiliary receptor* for TGFβ endoglin was significantly downregulated after losartan treatment. Endoglin is involved in angiogenesis and vessel homoeostasis; an *endoglin* gene mutation leads to hereditary hemorrhagic telangiectasia [27]. Endoglin regulates the expression and activation of endothelial nitric oxide synthase, which is believed to play a role in angiogenesis and in the composition of vascular resistance in aneurysm formation [28]. Endoglin plays a pivotal role in the differentiation of neural crest cells into the VSMC that populate the aorta. Mancini *et al.* demonstrated that in *endoglin ^−^/^−^* mice, defective VSMC development occurs, which leads to the assumption that endoglin plays an important role in vessel wall composition on aneurysm formation [29]. On the other hand, it has been shown that TGFβ and endoglin expressions are both elevated in cultured VSMCs derived from MFS patients. Losartan, an AT_1_R blocker, prevents aneurysm progression in MFS by decreasing TGFβ activity [30,31]. In our study, the expression of TGFβ and endoglin was decreased after treating a VSMC culture with losartan. This indicates the beneficial effects of losartan on the overexpression of TGFβ signaling pathway proteins. Whether the differences in TGFβ and endoglin levels in BAV patients are some of the causes of aneurysm development in BAV is unclear. Further investigations are warranted to clarify the effect of these proteins. A previous study showed direct dysregulation caspase-3 signaling in VSMCs isolated from BAV patients [20]. We are planning to induce apoptosis in primary VSMCs and to investigate the caspase-3 activity and signaling pathway in those cells in comparison with immortalized WG-59 cells. As the next step, we are planning on silencing important pathways (fibroblast growth factor, caspase-3 and endoglin [32]) for specific studies to improve our understanding of the mechanisms associated with apoptosis signaling in BAV.

## 5. Study Limitation

We are aware of the fact that we only present the generation of one cell line in this study and that multiple chromosomal abnormalities in our cell line may affect the results and limit the comparability of these cells with other primary cell culture. An update of the literature, however, scored no results regarding the generation of human VSMC culture isolated from BAV patients, enabling investigation of BAV ascending aorta.

Losartan should be metabolized to gain an activated form available for the organism. Telmisartan would be more suitable for *in vitro* usage. The application of telmisartan as an activated form of angiotensin II blocker, which on its part activates eNOS, will be one of our future projects. Telmisartan is known to decrease inflammatory protein expression, such as C- reactive protein in coronary plaque components [33]. However, according to our experience and previous results, only low inflammatory processes are detected in ascending aorta of BAV [34,35].

## Figures and Tables

**Table 1:** Karyotype of the examined immortalized cell culture showing several aberrations in different chromosomes. SKY, spectral karyotyping.

Composite Karyotype SKY:
70-91,XX,-Y[11],-Y[12],der(2)t(2;6)x2[11],-3[3],-4[4],der(6)t(6;15)[2],der(6)t(6;15der(8)t(9;8;2)x2[12],der(8)t(8;18)[7],der(8)t(8;18)x2[5],-9[4],+9[2],der(9)t(5;9)[5],der(9)t(5;9)x2[6],-10[4],-11[3],der(11)t(8;11)x2[7],der(12)t(5;12)x2[4],-13[7],-13[3],rob(13;14)[4],rob(13;14)x2[7],-14[5],-14[7],der(14)t(14,19)[4],der(14)t(14,19)x2[4],-15[12],-15[12], der(15)t(15;16)x2[9],-16[5], del(16)x2[8],der(16)t(16;20)x2[3],-17[4],-18[11],-18[7],der(18)t(5;18)[2],der(18)t(11;18)x2[7],-19[2]-19[10],-20[3],-20[2],der(20)t(20;22)x2[9],-21[3],-22[12],-22[12][cp12]

**Table 2:** Allelic list of the WG-59 cell line and tissue donor STR loci profiling using 8 different polymorph loci, performed by DSMZ, Germany, showing that these two samples are of the same origin.

Marker	WG-59 Cells	WG-59 Tissue
D5	10	10
D5’	12	12
D13	11	11
D13’	11	11
D7	9	9
D7’	10	10
D16	12	12
D16’	13	13
vWA	14	14
vWA’	15	15
TH01	9	9
TH01’	9.3	9.3
TPOX	8	8
TPOX’	11	11
CFS1	11	11
CSF1’	11	11
Amel	X	X
Amel’	X	Y

**Table 3:** Significantly regulated genes in WG-59 after losartan-treatment compared to untreated cells. −, downregulation; +, upregulation.

Gene	Description	Gene Bank	Fold Change	*t*-Test
			1 µM Losartan *vs*. Control	*p*-Value
*ACVR2*	Activin A receptor, type IIA	NM_001616.3	−1.16	0.0274
*BAMBI*	BMP and activin membrane-bound inhibitor	NM_012342.2	−1.10	0.0273
*BMP4*	Bone morphogenetic protein 4	NM_001202.3	−1.12	0.0325
*BMPER*	BMP binding endothelial regulator	NM_133468	−1.16	0.0121
*CDC25A*	Cell division cycle 25A	NM_001789.2	−1.24	0.0008
*CST3*	Cystatin C	NC_000020.10	−1.11	0.0305
*ENG*	Endoglin	NM_000118	−1.14	0.0052
*FST*	Follistatin	NM_013409	−1.13	0.0099
*GDF5*	Growth Differentiation Factor 5	NM_000557	−1.26	0.0091
*JUN*	Jun Proto-Oncogene	NM_002228	−1.06	0.0479
*PLAU*	Plasminogen Activator, urokinase	NM_002658	−1.07	0.0052
*SMAD3*	SMAD family member 3	NM_005902	1.01	0.0335
*SOX4*	SRY (sex determining region Y)-box 4	NM_003107	−1.25	0.0061
*STAT1*	Signal transducer and activator of transcription, 1.91 kD	NM_007315	−1.08	0.0015
*TGFB1*	Transforming growth factor beta 1	NM_000660	1.02	0.0460
*TGFBI*	Transforming growth factor, beta-induced, 68 kDa	NM_000358	−1.12	0.0316
*TGFBR1*	Transforming growth factor beta, receptor 1	NM_004612	−1.20	0.0028
*HPRT1*	Hypoxanthine phosphoribosyltransferase1	NM_000194	−1.04	0.0396
