# Erratum: Lazar-Karsten, P., et al. Generation and Characterization of Vascular Smooth Muscle Cell Lines Derived from a Patient with a Bicuspid Aortic Valve. *Cells* 2016, *5*, 19

**DOI:** 10.3390/cells5030036

**Published:** 2016-09-20

**Authors:** Pamela Lazar-Karsten, Gazanfer Belge, Detlev Schult-Badusche, Tim Focken, Arlo Radtke, Junfeng Yan, Pramod Ranabhat, Salah A. Mohamed

**Affiliations:** 1 Department of Cardiac and Thoracic Vascular Surgery, University of Luebeck, D-23538 Luebeck, Germany; pamela.lazar-karsten@uksh.de (P.L.-K.); detlev.schult-badusche@uksh.de (D.S.-B.); junfeng.yan@uksh.de (J.Y.); pramod.ranabhat@student.uni-luebeck.de (P.R.); 2 Center of Human Genetics and Genetic Counselling, University of Bremen, D-28359 Bremen, Germany; belge@uni-bremen.de (G.B.); focken@uni-bremen.de (T.F.); arlo.radtke@gmx.de (A.R.)

The authors wish to make the following erratum to this paper [1]. The author’s name “Pramod Renhabat” should be “Pramod Ranabhat”.

We apologize for any inconvenience caused to the readers.
